# *Sarcoptes *mite epidemiology and treatment in African buffalo (*Syncerus caffer*) calves captured for translocation from the Kafue game management area to game ranches

**DOI:** 10.1186/1746-6148-6-29

**Published:** 2010-06-01

**Authors:** Hetron M Munang'andu, Victor M Siamudaala, Wigganson Matandiko, Musso Munyeme, Mwelwa Chembensofu, Enala Mwase

**Affiliations:** 1 Department of Basic Sciences and Aquatic Medicine, Section of Aquatic Medicine and Nutrition, Norwegian School of Veterinary Sciences, Ullevålsveien 72, P. O. Box 8146 Dep, NO-0033 Oslo, Norway; 2 Zambia Wildlife Authority, P. O. Box 830124, Chilanga, Zambia; 3 School of Veterinary Medicine, University of Zambia, P.O. Box 32379, Lusaka, Zambia

## Abstract

**Background:**

In Zambia, translocation of wildlife from National Parks to private owned game ranches demands that only animals free of infectious diseases that could adversely affect the expansion of the wildlife industry should be translocated to game ranches. *Sarcoptes *mange (*Sarcoptes scarbiei*) has been involved in the reduction of wildlife populations in some species.

**Results:**

*Sarcoptes *mange (*Sarcoptes scarbiei*) was detected and eradicated from two herds of African buffalo (*Syncerus caffer*) calves captured in the Kafue GMA in July 2004 and August 2005. The overall prevalence was estimated at 89.5% (77/86). Sex had no influence on the occurrence and severity of the disease. Of the 86 calves used in the study, 72.1% had good body condition scores, 20.9% were fair and 7.0% were poor. Of the 77 infected calves, 53.2% were mildly infected, 28.6% were moderately and 18.2% were severely infected. Body condition score was correlated to the severity of the infection (r = 0.72, p < 0.000, *n *= 86) at capture. Eradication of *Sarcoptes *mites from the entire herd using ivermetcin was dependant on the severity of the infection. The overall ability of ivermectin to clear the infection after the first treatment was estimated at 81.8% (*n *= 77). It increased to 94.8% and 100% after the second and third treatments respectively.

**Conclusion:**

This is the first report on the epidemiology and treatment of *Sarcoptes *mange in African buffaloes in Zambia. This study improves our understanding about *Sarcoptes scabiei *epidemiology and treatment which will have further applications for the safe animal translocation.

## Background

Sarcoptic mange is a highly contagious disease affecting humans [1,2], domestic animals [3] and wildlife [4]. The disease is caused by *Sarcoptes scabiei *mites that burrow in the epidermis of the skin where they form tunnels and deposit their eggs and digestive secretions causing intense skin irritations. The mites complete their lifecycle within a few weeks giving rise to high densities reaching up to 5000 mites/cm^2 ^in some species [5]. This leads to alopecia and hyperkeratosis of the skin [6]. The disease has been associated with high morbidity and mortality leading to population reductions in some species [7-10]. The efficacy of ivermectin in the treatment of *Sarcoptes *mange in different wildlife species has been carried out with variable responses [11-14]. Although the disease affects various wildlife species [15,15], there are few reports describing its epidemiology in African game. In Zambia, it has been reported in humans and domestic animals with limited reports on wildlife [16].

The expansion of the game ranching industry in Zambia as an *ex-situ *conservation strategy has led to the translocation of several wildlife species from state owned National Parks (NPs) and game management areas (GMAs) to private owned game ranches in recent years. However, infectious diseases such as foot and mouth disease, *Sarcoptes *mange and bovine tuberculosis are a major constraint to the translocation of wildlife [17] because regulations demand that only animals not infected with such infectious diseases should be translocated to game ranches. This paper discusses the clinical observations made and the treatment regime used to eradicate sarcoptic mange from the entire herd of buffalo calves captured in the Kafue GMA prior to translocation.

## Methods

### Animal data

The Study Was Carried Out At Nanzhila (26°03'E, 15°44'S) located on the western end of the Kafue flats GMA which is a wetland area designated as Ramsar Site No. 530 [18]. The area supports a transhumance grazing system for the traditional livestock farmers from the surrounding upland areas by providing grazing pastures for cattle in the dry season. GMAs are considered to be buffer zones being located between NPs and communal lands and are also referred to as livestock/wildlife interface areas because the co-existence of domestic animals and wildlife is permitted in these areas [19]. In August 2004 a total of 48 African buffalo calves were captured and used in the study while 38 were used in July 2005 with permission of the Zambia wildlife Authority (ZAWA) who are the custodians of wildlife in Zambia (Project authorization ref: 006/68-07-05). Age was estimated at four to six months for all calves using tooth development [20]. The calves were kept in quarantine facilities for 90 days and were sampled at 30 days intervals. At every sampling, calves were immobilized using M99 (etorphine hydrochloride, Novartis Ltd., Animal Health, South Africa) and reversed using M5050 revivon (dreprenorphine, Novartis SA Ltd., Animal Health, South Africa).

#### *Sarcoptes *diagnosis and treatment

The severity of the disease was classified into three categories as mild (+), moderate (++) and severe (+++) as described by Pence *et al *[21]. The mild group involved calves with focal lesions covering small patches of the body surface. Most of the lesions observed in this group were in the initial phase and they included erythema, pruritis, encrustations, suppuration and alopecia. The most affected body parts were the forehead, shoulders, hump and hock joints. The moderate group involved animals with actively progressing lesions covering large areas extending to almost half of the body surface. There were large surfaces of erythema, pruritis, alopecia, scabs, parakeratosis and fissures on some of the animals. Suffice to mention that some of the lesions observed in this group were similar to those observed in the mild group only that in the moderate group lesions were progressive and diffuse covering large areas. Severe groups were animals whose lesions covered more than half of the body surface. Alopecia, parakeratosis, thick and wrinkled skin surfaces were more pronounced in this group than in the mild and moderate groups. Body condition score was recorded as good (++), fair (+) or poor (-) depending on the degree of emaciation and skin pliability. Highly emaciated animals having little muscle with no sign of fat deposits and the skin falling slowly or staying in a fold after pulling a portion on the neck were scored poor (-). Animals having moderate deposition of muscle with the skin of the neck folding moderately faster than those in poor body conditions were scored as fair (+) while animals with excess fat and muscle with the skin quickly falling to its original position after the pliability test were scored as good (++).

Skin scrapings were collected at every sampling. All claves were treated with 200 μg kg^-1 ^ivermectin (Novartis SA Ltd., Animal Health) at each sampling time point irrespective of whether they had mites detected or not. They were translocated to game ranches within three days after being declared free of mites after the fourth screening test.

At the School of Veterinary Medicine at the University of Zambia, skin scrapings were examined after treatment with 20% potassium hydroxide [22]. Identification of mites was carried out as described elsewhere [23]. Sex, age, body condition score, severity score of the clinical condition and laboratory results for each calf were recorded in Exel^® ^sheets at each sampling time point. Data was analyzed using STATA SE/10 for windows (Stata Corp. College Station, TX, USA, http://www.stata.com). Chi-square was used to test the measures of association between sex and occurrence of the disease while the Pearson's correlation coefficient test was used to measure the correlation between severity of the disease and body condition score.

## Results

### *Sarcoptes *mite epidemiology

Sex ratio (male:female) was estimated at 1:12. Our observations indicate that sex had no influence on the occurrence (χ^2 ^= 0.093, p > 0.70) and severity (χ^2 ^= 0.70, p > 0.70) of the disease. Overall prevalence for the two herds was estimated at 89.5% (77/86) at capture (Table 1). Of the 86 calves used in the study, 72.1% had good body condition score, 20.9% were fair and 6.9% were poor at capture (Table 2). Of the 77 infected calves, 53.2% were mildly infected, 28.6% were moderately and 18.2% were severely infected at capture. Body condition score was positively correlated to the severity of the infection (r = 0.72, p < 0.000, *n *= 86) at capture.

**Table 1:** Treatment regime and detection of mites at different sampling time points.

				Number of calves detected with presence of *Sarcoptes scabiei *mites at;			
				
Year	Number of calves	Severity of skin infection	Severity score	1st Sampling and treatment (Day 1)	2nd Sampling and treatment (Day 30)	3rd sampling and treatment (Day 60)	4th sampling (Day 90)
2004	48(45)*	Mild	+	25	2	0	0
		Moderate	++	12	4	0	0
		Severe	+++	8	6	2	0
2005	38(32)*	Mild	+	16	0	0	0
		Moderate	++	10	4	0	0
		Severe	+++	6	4	2	0

Totals	86(77)*			77	14	4	0

*Number of calves infected with *Sarcoptes *mange at capture (first sampling).

**Table 2:** Body condition score of the calves at different sampling time points.

				Body score condition at different sampling time points			
				
Year	*n*	Category	Score	Day 1	Day 30	Day 60	Day 90
							
				Number of calves	Number of calves	Number of calves	Number of calves
2004	48	Good	++	35	47	48	48
		Fair	+	11	1	-	-
		Poor	-	2	-	-	-
2005	38	Good	++	27	36	38	38
		Fair	+	7	2	-	-
		Poor	-	4	-	-	-

Totals	86			86	86	86	86

### *Sarcoptes *mite treatment

Decrease in the number of infected calves after the first treatment was related to the severity of the infection observed at capture (Table 1). Of the 41 mildly infected calves, only two had *Sarcoptes *mites after the first treatment (4.98%) while in the moderately affected group 45.45% (10/22) needed a second treatment for the mites to be eliminated. In the severely affected group, there was a gradual decline with 28.57% (4/14) not having mites after the first treatment, 42.86% (10/14) after the second treatment and another 28.57% (4/14) after the third treatment. The overall drug ability to clear the infection was estimated at 81.8% (*n *= 77) after the first treatment. It increased to 94.8% after the second treatment and reached 100% after the third treatment. The calves were translocated to game ranches within three days after the fourth treatment after they were declared free of *Sarcoptes *mange.

## Discussion

In Zambia, sarcoptic mange has been reported in sitatunga (*Tragelaphus spekii*), hartebeest (*Alcephelus lichtensteini*) and silver-backed-jackal (*Canis mesomelas*) in wildlife [16]. In domestic animals the disease has been reported in cattle, goats and pigs [16]. It has not been reported from the African buffalo and no comprehensive study has been carried out describing the clinical findings in wildlife in Zambia. In line with observations made by Pence and Ueckermann [24], our findings indicate that clinical conditions vary between animals depending on the severity of the infection. Tikaram and Ruprah [25]observed that the incidence of sarcoptic mange was highest in young animals below one year. They noted that prevalence decreased with advancement of age and that sex had no influence on the incidence of the disease [25]. The high prevalence 89.5%(77/86) obtained in the present study could be attributed to the fact that the animals involved were young ranging between four and six months of age at capture. We also observed that sex had no influence on the occurrence of the disease in the calves.

Fain *et al *[26] postulated that *Sarcoptes *mange originated as a human parasite that spread to domestic animals and subsequently transmitted to various wildlife species by contact with their domestic counterparts. It is not known how *Sarcoptes scabiei *was introduced in buffalo herds in the Kafue GMA although the disease has been present in domestic animals in the area [16]. Studies have shown that the transmission of mange was density dependant being more prevalent during times of higher environmental stress such as drought [27,28]. In the present study, the high prevalence of *Sarcoptes *mange (89.5%, *n *= 86) reported in the calves could have been exacerbated by stress factors caused by the scarcity of water and grazing pastures during the dry season in the months of May to October on the Kafue flats. Livestock owners in the upland areas surrounding the Kafue flats practice a transhumance grazing system by moving their animals into the flats during the dry season leading to the sharing of grazing pastures and water between wildlife and livestock. This impacts a high population density on the flats. It is likely that *Sarcoptes *mange could have been transmitted from livestock to wildlife during such contacts. However, to fully understand the factors responsible for the epidemiology and transmission of the disease between species in the Kafue flats it is imperative that wildlife and livestock surveys be synchronized involving molecular studies of the gene flow of this parasite between different host species and geographical localities [29]. Perez *et al *[30] observed that *Sarcoptes *mange cases were higher in the cold season reducing in the hot months in summer when conditions were unfavorable for the survival of the mites. In Zambia, the cold season is in the months of June to August and this could have contributed to the increase in the number of calves infected given the timing of the present study that calves were captured in the cold months of August 2005 and July 2005. Treating the calves for the subsequent three months means that the treatment regime extended into the hot months of September to November which could have impacted negatively on the survival of the mites. It is likely that climatic factors had confounding effects on the treatment regime used in the current study. However, there is a need to carry out more detailed studies in order to determine the effect of climate on the epidemiology of sarcoptic mange on the Kafue basin.

The efficacy of ivermectin in the treatment of *Sarcoptes *mange in domestic animal has been studied by different scientists with some studies showing high responses after the first treatment [31,32] although other studies have shown a gradual decrease in the number of mites after multiple doses of ivermetcin administered at regular intervals [33]. In Wildlife, there have been variable responses on the treatment of *Sarcoptes *mange using ivermectin in different animal species. Leon-Vizcaino *et al *[34] managed to eliminate the *Sarcoptes *mites from Spanish ibex (*Capra pyrenaica*) in the early stages of the infection, although double injections with high doses of ivermectin did not yield success in chronically infected animals. However, Skerratt [35] reported a complete resolution of clinical signs in wombats (*Wombatus ursinus*) after three injections of ivermectin administered 10 days apart although mites were not completely eliminated until the wambots were given a second regime of treatment. Yeruham *et al *[36] reported of complete recovery of severely infected wild ruminants kept in zoological gardens after mutlitple treament using ivermectin administered orally at regular intervals. These observations indicate that *Sarcoptes *mites can be eliminated from wild animals kept in captivity using ivermectin. Besides, ivermectin has been shown to be an effective drug for treating various endoparasites and parasitic skin diseases [37]. In the present study, we observed a gradual decrease in the number of infected calves from the first to the third treatment (Table 1). Our observations indicate that eradication of *Sarcoptes scabiei *mites using ivermectin was dependant on the severity of the infection (Table 1) similar to observations made by Skerratt [35].

## Conclusion

*Sarcoptes *mites were eradicated from African buffalo calves captured from the Kafue game management area after the third treatment indicating that it is possible to eradicate some infectious diseases from infected wildlife populations prior to translocation by using the screening and treatment method. Hence, the risk of introducing *Sarcoptes *mange to naïve wildlife populations on the game ranches was avoided. Suffice to mention that such disease eradication methods are expensive as they require frequent immobilization of animals. Prolonged keeping of wildlife in captivity imposes a lot of stress on the animals. Therefore, we recommend that highly potent drugs aimed at reducing the need for repeat treatments should be explored for use in wildlife medicine. In addition, disease control strategies involving less strenuous handling procedures should be used with a view of promoting the success of *ex-situ *conservation.

## Authors' contributions

HMM and VS conceived the study, participated in designing, coordination and capture of the animals. They were instrumental in drafting the manuscript. WM and MM participated in the capture and sampling as well as in drafting the manuscript. MC and EM carried out the laboratory diagnosis. All authors read and approved the manuscript.
